# Microbiota profiling reveals alteration of gut microbial neurotransmitters in a mouse model of autism-associated 16p11.2 microduplication

**DOI:** 10.3389/fmicb.2024.1331130

**Published:** 2024-03-26

**Authors:** Zhang Fu, Xiuyan Yang, Youheng Jiang, Xinliang Mao, Hualin Liu, Yanming Yang, Jia Chen, Zhumei Chen, Huiliang Li, Xue-Song Zhang, Xinjun Mao, Ningning Li, Dilong Wang, Jian Jiang

**Affiliations:** ^1^Tomas Lindhal Nobel Laureate Laboratory, The Seventh Affiliated Hospital of Sun Yat-sen University, Shenzhen, Guangdong, China; ^2^Digestive Diseases Center, Guangdong Provincial Key Laboratory of Digestive Cancer Research, The Seventh Affiliated Hospital of Sun Yat-sen University, Shenzhen, Guangdong, China; ^3^Guangdong Perfect Life Health Science and Technology Research Institute Co., Ltd., Zhongshan, Guangdong, China; ^4^Department of Anesthesiology, The Seventh Affiliated Hospital of Sun Yat-Sen University (SYSU), Shenzhen, Guangdong, China; ^5^Division of Medicine, Wolfson Institute for Biomedical Research, Faculty of Medical Sciences, University College London, London, United Kingdom; ^6^China-UK Institute for Frontier Science, Shenzhen, Guangdong, China; ^7^Center for Advanced Biotechnology and Medicine, Rutgers University, Piscataway, NJ, United States; ^8^Department of Anesthesiology, The Affiliated Hospital of Youjiang Medical University for Nationalities, Baise, Guangxi, China; ^9^Department of Pediatrics, Sun Yat-sen Memorial Hospital, Sun Yat-sen University, Guangzhou, Guangdong, China

**Keywords:** ASD, 16p11.2, gut microbiota, 16S rRNA, metabolomic, histamine, neurotransmitter

## Abstract

The gut-brain axis is evident in modulating neuropsychiatric diseases including autism spectrum disorder (ASD). Chromosomal 16p11.2 microduplication 16p11.2^dp/+^ is among the most prevalent genetic copy number variations (CNV) linked with ASD. However, the implications of gut microbiota status underlying the development of ASD-like impairments induced by 16p11.2^dp/+^ remains unclear. To address this, we initially investigated a mouse model of 16p11.2^dp/+^, which exhibits social novelty deficit and repetitive behavior characteristic of ASD. Subsequently, we conducted a comparative analysis of the gut microbial community and metabolomic profiles between 16p11.2^dp/+^ and their wild-type counterparts using 16S rRNA sequencing and liquid chromatography-mass spectrometry (LC/MS). Our microbiota analysis revealed structural dysbiosis in 16p11.2^dp/+^ mice, characterized by reduced biodiversity and alterations in species abundance, as indicated by α/β-diversity analysis. Specifically, we observed reduced relative abundances of *Faecalibaculum* and *Romboutsia*, accompanied by an increase in *Turicibacter* and *Prevotellaceae UCG_001* in 16p11.2^dp/+^ group. Metabolomic analysis identified 19 significantly altered metabolites and unveiled enriched amino acid metabolism pathways. Notably, a disruption in the predominantly histamine-centered neurotransmitter network was observed in 16p11.2^dp/+^ mice. Collectively, our findings delineate potential alterations and correlations among the gut microbiota and microbial neurotransmitters in 16p11.2^dp/+^ mice, providing new insights into the pathogenesis of and treatment for 16p11.2 CNV-associated ASD.

## Introduction

The gut microbiota comprises an intricate community of microorganisms residing in the gastrointestinal tract (McCallum and Tropini, 2023). Over the past decade, scientific research has shed light on a robust bidirectional communication network linking the gastrointestinal system and the central nervous system, commonly acknowledged as the microbiota-gut-brain axis (Agirman and Hsiao, 2021). Disruptions in the gut microbiome and their consequent influence on the gut-brain axis surfaced as significant factors in the onset of neurodevelopmental disorders, such as Autism spectrum disorder (ASD) (Wang Q. et al., 2023), Parkinson’s disease (Nie and Ge, 2023), depression (Donoso et al., 2023), and Alzheimer’s disease (Zhu et al., 2023).

ASD is an immensely varied neurodevelopmental condition typified by recurrent stereotypical behaviors and impairments in social interaction (Weiss et al., 2008). Recent epidemiological findings indicate a prevalence rate of 2.3% among children and adolescents in the United States in 2018 (Li et al., 2022). Notably, individuals diagnosed with ASD frequently manifest gastrointestinal symptoms, including diarrhea, constipation, and gaseousness, alongside the core features of the disorder (Fulceri et al., 2016; Restrepo et al., 2020). Recent investigations have underscored the substantial influence of the gut microbiota on both gastrointestinal manifestations and neurodevelopmental aspects in individuals with ASD. Intestinal microorganisms have the capacity to synthesize neurotransmitters, thereby impacting the microbiota-gut-brain axis (Srikantha and Mohajeri, 2019). Specific microbial species, such as commensal bacteria *Bacteroides vulgatus*, *Eggerthella lenta*, and *Clostridium botulinum*, could influence the onset and development of ASD by altering glutamate-glutamine metabolism, reducing cortisol, and disrupting the metabolism of aromatic amino acids (Wang et al., 2019). Notably, previous studies revealed the relation of the pathogenic bacterium *Clostridium botulinum* to gastrointestinal symptoms in ASD (Ding et al., 2017) and discussed its potential role in promoting ASD through secreted toxins, which affect the expression of the GTPase RHO family (Argou-Cardozo and Zeidan-Chulia, 2018), although the difference in toxicology leading to fatal disease versus ASD remains unclear. Furthermore, fetal histamine concentrations have been linked to the presence of diarrhea symptoms in individuals displaying elevated IFN-γ levels within the ASD cohort (Xu et al., 2023). Moreover, disruptions in the gut microbiota may disturb neurotransmitter regulation, potentially contributing to the development of neurodevelopmental disorders. For example, anthocyanins have been demonstrated to increase the abundance of *Lactobaccillales*, stimulating enterochromaffin (EC) cells, enhancing the availability of tryptophan for serotonin synthesis. This effect leads to improved social interactions and reduced repetitive behaviors in mice with ASD induced by valproic acid exposure (Serra et al., 2022).

Within individuals diagnosed with ASD, one of the most prevalent genetic variations is the 16p11.2 copy number variants (CNVs), affecting approximately 1% of ASD cases (Kumar et al., 2008; Marshall et al., 2008). This genetic locus spans approximately 600 kb and encompasses 29 protein-coding genes. Microduplications or microdeletions within this region have been linked to a spectrum of disorders, including neurological conditions like ASD (Fernandez et al., 2010) and schizophrenia (Chang et al., 2021), as well as metabolic irregularities, hematological conditions, skeletal anomalies, genitourinary abnormalities, and sleep disturbances (Wu et al., 2015; Verbitsky et al., 2018; Crawford et al., 2019; Giannuzzi et al., 2019; Kamara et al., 2021; Wang et al., 2022; Hanssen et al., 2023). Nevertheless, the precise mechanisms by which 16p11.2 CNVs influences the onset of ASD remain inadequately understood (Rein and Yan, 2020). Autism is a multifaceted disorder, with genetic, environmental, and gut microbiota factors all at play (Sauer et al., 2021). It’s crucial to understand how the 16p11.2 genetic variant influences autism’s onset and/or progression, not just through genetic mechanisms, but also through non-genetic factors. While research has been conducted revolving around 16p11.2 microdeletion syndrom and gut microbiota, the specific effects of 16p11.2 microduplications on gut microbiota, and how these changes might relate to autistic symptom, are still unclear.

The 16p11.2^dp/+^ mouse model characterized by a microduplication within the 7Slx1b-Sept1 region, provides an effective tool for investigating the functional implications of 16p11.2^dp/+^. To explore the intricate and multifaceted connections between 16p11.2^dp/+^ and gut microbiota within the context of ASD-like symptoms, we initially confirmed the presence of core ASD-related behaviors in 16p11.2^dp/+^ mice. Following this, we conducted an analysis of the gut microbiota and metabolites in fecal samples collected from 16p11.2^dp/+^ mice and wild-type (WT) mice, using 16S rRNA sequencing and liquid chromatography-mass spectrometry (LC/MS). Our 16S rRNA data generated a total of 915 amplicon sequence variants (ASVs) from 16p11.2^dp/+^ mice and 10 WT mice. Additionally, we performed a metabolomics assessment, identifying a total of 458 metabolites in these samples. Furthermore, we conducted a correlation analysis between fecal metabolites and individual bacteria to explore the relationships between these distinct microbial species and fecal metabolites.

## Materials and methods

### Animal

16p11.2^dp/+^ mice, characterized by a heterozygous duplication of mouse chromosome 7F3 that is syntenic to the human 16p11.2 locus, were procured from Jackson Laboratory (Stock No: 013129). These mice were then crossbred with WT C57BL/6 background female mice (Horev et al., 2011). To maintain the colony, breeding involved crosses between WT females and 16p11.2^dp/+^ males. For the control group, offspring of WT males were employed. A total of 10–11 mice per group were involved in the study. All mice were provided with sterile food and autoclaved water *ad libitum* and subjected to a 12-h light cycle. The study was conducted in strict adherence to the ethical guidelines and protocols outlined by the Institutional Animal Care and Use Committee at Southern University of Science and Technology.

### Behavioral testing

#### Three-chamber test

The social behavior test was adapted from experiments previously described (Sgritta et al., 2019). The three-chamber apparatus consisted of a rectangular box (60 × 40 × 20 cm, L × W × H) divided into three interconnected chambers. Mice were habituated for 10 min in the empty apparatus. Sociability was then assessed over a 10-min period during which the mice had the option to interact with either an empty cage or a stranger mouse (M). Subsequently, preference for social novelty was tested for 10 min by introducing another stranger mouse (Nov). The time spent by the test mouse interacting with the empty cage, Fam mouse or Nov mouse was recorded and measured using the EthoVision XT 10 software package. The human observers were blind to the treatment group.

#### Open field test

The open field test was adapted from experiments we previously described (Jiang et al., 2022). Mice were placed in a box (40 × 40 × 40 cm^3^) and allowed to explore freely for 10 min. The interior 20 × 20 cm^2^ area was defined as center area. Distance traveled, velocity of movement, and duration in the center area were recorded and automatically analyzed by Noldus EthoVision XT10 (Noldus Information Technology; Leesburg, VA, United States).

#### Self-grooming test

The mice were placed in an empty cage containing corncob bedding for 5 minutes to acclimate. Each mouse then recorded how long it spent grooming itself over the next 10 minutes. Self-grooming includes wiping the face, scratching/rubbing the head and ears, and grooming the whole body.

#### Elevated plus maze

The maze was composed of two open and two closed arms intersecting each other, elevated 1 m above the floor. The Elevated plus maze was equipped with a video tracking system, and the videos were analyzed by Noldus EthoVision XT10 software. The test mice were placed in the center facing the open arm, and their activity was measured for 5 min. The total time spent in open arms, center area and closed arms was recorded.

#### Fecal sample collection

After a feeding period of 90 days, we collected fresh fecal samples from both 16p11.2^dp/+^ male mice and their WT counterparts. For each mouse, we collected at least two tubes of samples, with each tube containing approximately 200 mg of fecal matter.

#### DNA extraction and 16S rRNA sequencing

Genomic DNA was isolated from fecal samples of mice utilizing the hexadecyltrimethylammonium bromide (CTAB) technique, as outlined in the study conducted by the pervious study (Kang et al., 2019). In brief, we extracted total microbial DNA of superior quality using the E.Z.N.A Stool DNA Kit (manufactured by Omega Bio-Tek, Inc., Norcross, GA, United States), adhering to the guidelines provided by the manufacturer. The amplification of the V3-V4 regions of the 16S rRNA gene was carried out using PCR, involving an initial step at 98°C for 1 min, followed by 30 cycles of denaturation at 98°C for 10 s, annealing at 50°C for 30 s, elongation at 72°C for 60 s, and a final extension step at 72°C for 5 min. Universal bacterial primers, 341F (5’-CCTAYGGGRBGCASCAG-3′) and 806R (5’-GGACTACNNGGGTATCTAAT-3′) were used for this purpose. For the preparation of sequencing libraries, the NEB Next®Ultra™DNA Library Prep Kit for Illumina (NEB, USA) was employed following the manufacturer’s guidelines, and index codes were incorporated. Library quality was assessed using the Qubit@ 2.0 Fluorometer (Thermo Scientific) and the Agilent Bioanalyzer 2,100 system. Ultimately, the library underwent sequencing on a HiSeq2500 PE250 platform, generating paired-end reads of 250 base pairs.

#### Fecal metabolomic analysis

Metabolites in stool samples were determined by LC-MS. Briefly, 800 μL of a methanol-acetonitrile solution (1:1, v/v) was added to 80 mg of fecal sample, vortexed, and then centrifuged. The samples were incubated on ice for 10 min and centrifuged at 14,000 rpm at 4°C for 20 min, after which the supernatant was retained and stored at −80°C. For Ultra High Performance Liquid Chromatography (UHPLC)-MS/MS analysis, we used an Agilent 1,290 Infinity LC system (Agilent, 1.7 μm, 2.1 mm × 100 mm) to perform chromatographic separation of the samples at a constant temperature of 25°C and an AB Triple TOF 6600 series mass spectrometer (AB SCIEX) to detect eluted metabolites. The raw data files were converted into .mzXML format by ProteoWizard, and then the XCMS program^1^ was used to perform peak alignment and quantification for each metabolite. Subsequently, peak intensities were normalized to the total spectral intensity.

### Data analysis and statistic tests

The data from behavioral tests for the two groups underwent t-test for analysis. In the case of the three-chamber test results, a two-way ANOVA was employed, followed by Bonferroni’s multiple-comparisons test for further analysis.

The standard amplicon sequencing data analyses were carried out using QIIME 2 and its DADA2 plugin. These analyses included sequence quality control, the generation of feature tables and representative sequences, taxonomy assignment of representative sequences against the SILVA SSURef NR99 version 138.1 database, and the construction of phylogenetic trees. α-diversity and β-diversity analyses were performed using the R package vegan. To assess the significant differences in α-diversity between 16p11.2^dp/+^ and WT mice, the Tukey HSD test integrated into the alpha_boxplot function of the R package amplicon was utilized. Principal Coordinates Analysis (PCoA) was applied based on the Bray-Curtis distance using the R package vegan to identify the community compositions in all samples. The analysis of differential taxa was conducted using LEfSe. KEGG pathway predictions were performed using PICRUSt2. The differential abundance of phylum, genus, and functional modules between any two groups was assessed using the Wilcoxon rank-sum test. The *p*-values were adjusted for multiple testing with the Benjamini-Hochberg method to control the false discovery rate (FDR).

Partial least squares discriminant analysis (PLS-DA) and orthogonal partial least-squares discriminant analysis (OPLS-DA) were employed to visualize and compare the metabolites in each group, with metabolic variations between the groups detected using a permutation testing algorithm. Significant differential metabolites were identified using a threshold of *p* value (t-test) < 0.05 and VIP > 1, and clustering blot maps were created. The correlation between microbes and metabolites was analyzed using Spearman correlation analysis, with significance set at *p* < 0.05.

## Results

### 16p11.2^dp/+^ mice exhibit social novelty deficits and repetitive behaviors

**Figure 1:**
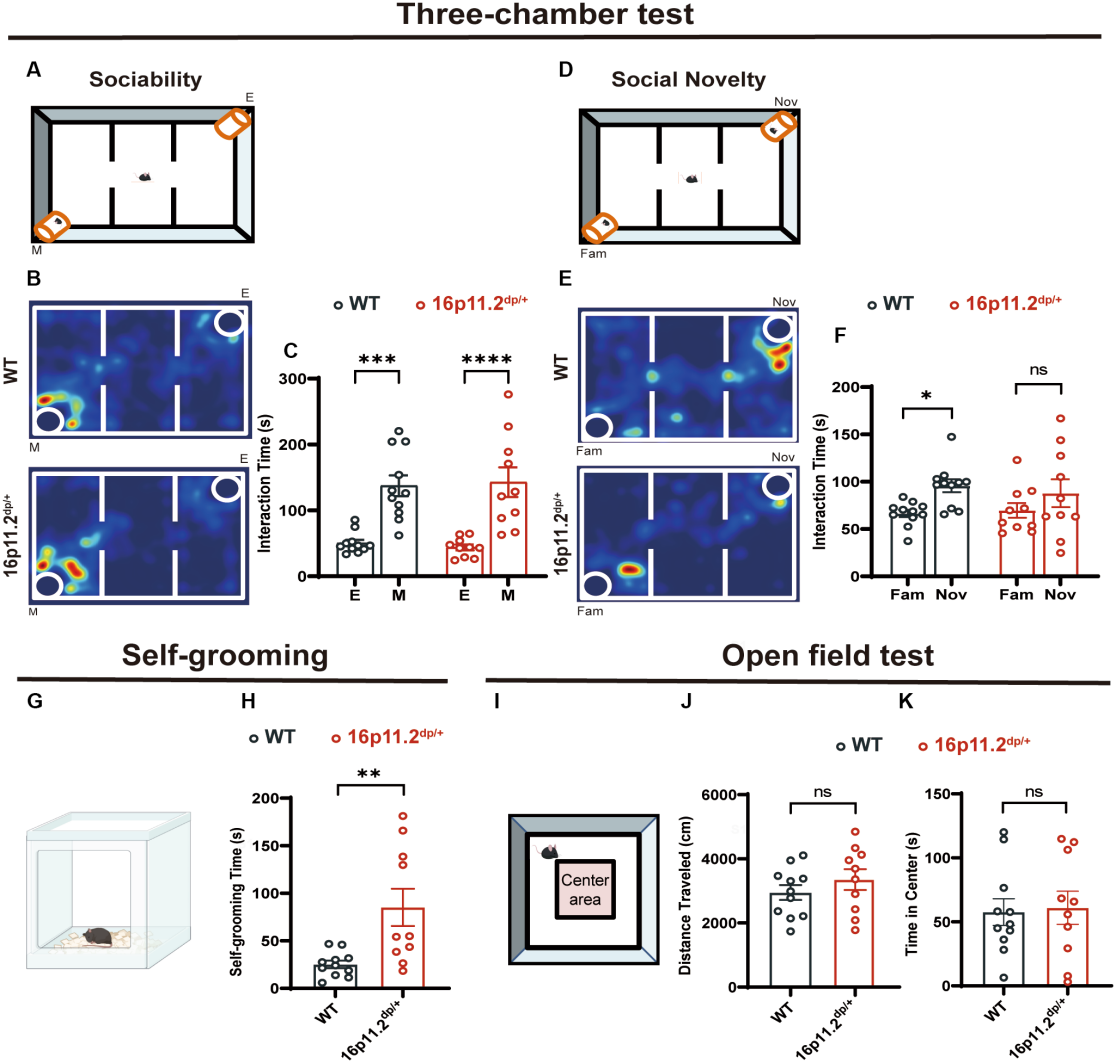
16p11.2^dp/+^ mice showed social novelty deficits and repetitive behaviors. **(A,D)** Illustrations of the three-chamber test (E: empty cage; M: stranger mouse; Fam: Familiar mouse; Nov: novel stranger mouse). **(B)** Representational heat maps during the sociability phase. **(C)** The duration spent interacting with a mouse (social) and an empty cage (non-social) during 10 min. **(E)** Representational heat maps during the social novelty phase. **(F)** 16p11.2^dp/+^ mice displayed reduced preference for the novel social mouse compared to WT mice. **(G)** Illustration of the mouse self-grooming experiment. **(H)** Self-grooming time in 16p11.2^dp/+^ mice was significantly increased. **(I)** Diagram representing the open field test. **(J,K)** No significant differences were observed in the distance traveled and time spent in the central area between 16p11.2^dp/+^ mice and WT mice. Statistical significance is indicated as follows: Data is presented as mean ± SEM. ^*^p < 0.05, ^***^p < 0.001, ^****^p < 0.001, ns: not significant, WT: n = 11; 16p11.2^dp/+^: n = 10.

To investigate if 16p11.2^dp/+^ mice recapitulate clinical characteristics seen in humans, we conducted behavioral tests on 8-10-week-old male 16p11.2^dp/+^ mice and age-matched WT mice. We tested ASD-related behaviors such as social skills, repetitive behaviors, and anxious behaviors. In the three-chamber test, assessing sociability and social recognition, both 16p11.2^dp/+^ and WT mice showed a preference for a chamber with a stranger mouse (M) over an empty cage (E) (Figures 1A–C). Interestingly, during the social novelty phase, the 16p11.2^dp/+^ mice exhibited a significantly lower preference for novel mice (Nov) compared to that of WT mice. This suggests a potential defect in social novelty preference in 16p11.2^dp/+^ mice (Figures 1D–F). In addition to social deficits, repetitive behaviors are another core symptom of ASD. To assess the repetitive behaviors of 16p11.2^dp/+^ mice, we observed the frequency of grooming within a 10-minute period in their home cage. Notably, 16p11.2^dp/+^ mice engaged significantly more in self-grooming, an indicative repetitive behavior (Figures 1G,H). Given that individuals with ASD often exhibit certain anxiety-like behaviors, we sought to test whether 16p11.2^dp/+^ mice display similar behaviors. We measured motor and anxiety-like behavior in mice through open field tests (Figures 1I–K) and an elevated plus maze (Supplementary Figure S1). However, these experiments did not reveal any significant anxiety-like behavior in 16p11.2^dp/+^ mice. In summary, our findings indicate that 16p11.2^dp/+^ mice display deficits in social novelty recognition and engage in stereotyped repetitive behaviors reminiscent of ASD.

### Altered gut microbiota in 16p11.2^dp/+^ mice compared to WT mice

**Figure 2:**
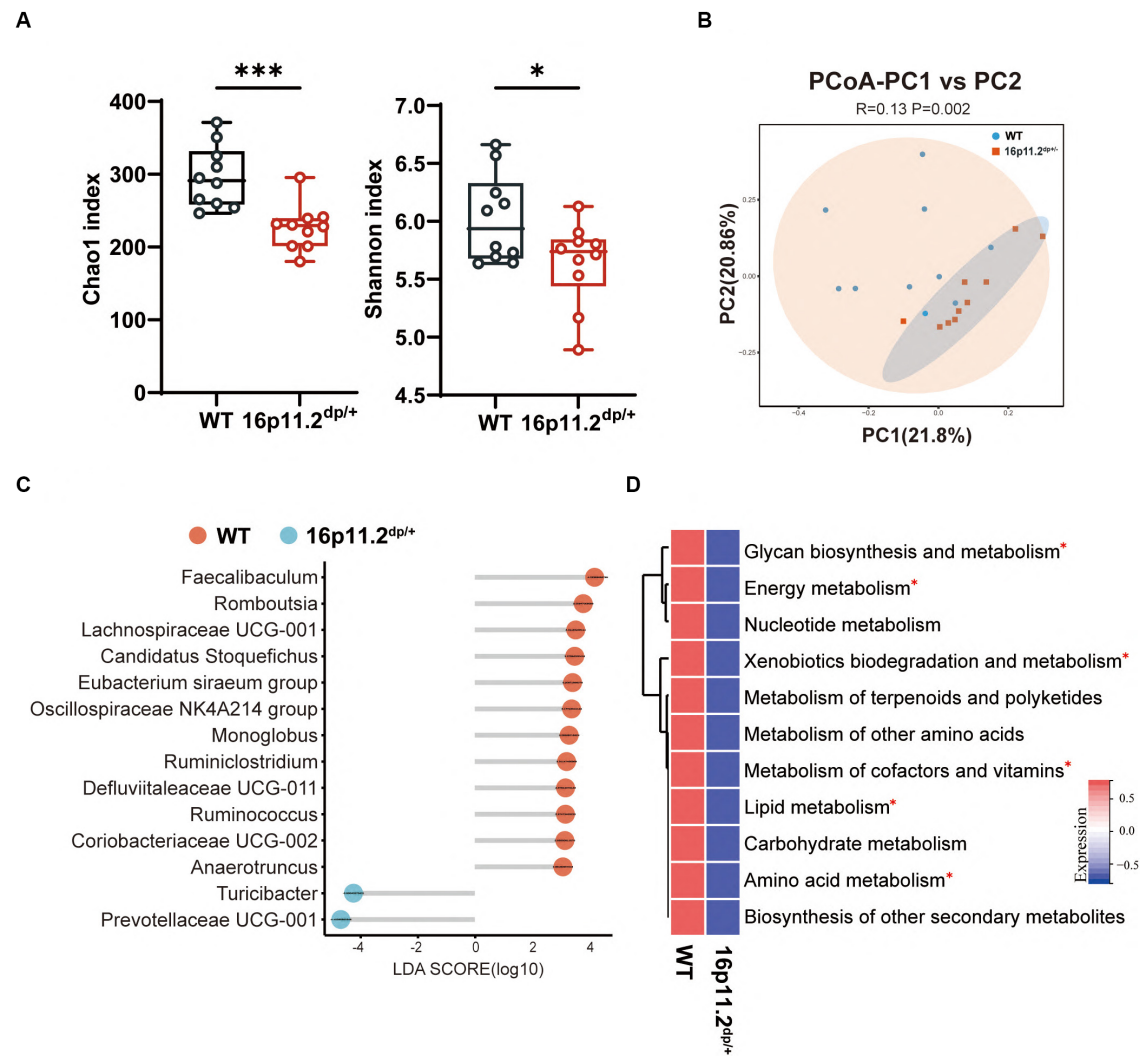
Analysis of microbial composition and functional implication in 16p11.2^dp/+^ mice versus WT mice. **(A)** Alpha diversity metrics, including Chao1 and Shannon indices, were calculated using fecal samples collected from 16p11.2^dp/+^ mice and WT mice. t-test, ^*^p < 0.05, ^***^p < 0.001. **(B)** Beta diversity was assessed through a principal coordinates analysis (PCoA) plot comparing the 16p11.2^dp/+^ and WT groups. PERMANOVA was used, *p* < 0.05. **(C)** Linear Discriminant Analysis (LDA) scores for differentially abundant bacterial taxa at the genus level between 16p11.2^dp/+^ and WT groups (LDA > 2, *p* < 0.05, FDR < 0.05). Red bars represent taxa enriched in WT group, while green bars represent taxa enriched in 16p11.2^dp/+^ group. **(D)** Average abundance of KEGG pathways that were differentially enriched in 16p11.2^dp/+^ and WT mice at the level 2. Significance indicated by t-test, *FDR < 0.05. All experimental sections are based on analyses of 10 mice per group.

To unearth possible gut microbiota distinctions in 16p11.2^dp/+^ mice versus their WT counterparts, potentially underpinning the observed behavioral defects, we conducted 16S rRNA sequencing. A total of 919 amplicon sequence variants (ASVs) were identified, with 915 remaining after the removal of 4 that were unassigned to bacteria. As depicted in Figure 2A, the Chao1 index and Shannon index were significantly lower in the 16p11.2^dp/+^ group compared to the WT group. The same trend was also observed in other α-diversity estimation methods, including ACE index (Supplementary Figure S2A), and richness (Supplementary Figure S2B). The results suggest that the 16p11.2^dp/+^ mice exhibited lower biodiversity and species abundance in comparison to the WT mice. A PCoA was conducted to assess the degree of similarity between microbial communities in the two groups using Bray–Curtis distance metrics (Figure 2B). The analysis revealed that the microbiota composition of the 16p11.2^dp/+^ group exhibited significant distinctions from that of the WT group. We performed a comprehensive analysis of bacterial composition at multiple taxonomic levels. The 16p11.2^dp/+^ mice and WT mice displayed different microbial profiles at both the phylum and genus levels (Supplementary Figures S2D,E). Notably, compared to the WT group, the 16p11.2^dp/+^ group was characterized by higher *Bacteroidetes* levels and a decreasing trend in the *Firmicutes*/*Bacteroidetes* ratio, although this trend did not reach statistical significance (Supplementary Figure S2C).

Next, we employed the LEfSe method to examine genus-level differences in gut microbiota between two groups of mice (LDA score > 2.0, *p* < 0.05). The lollipop plot unveiled a noteworthy alteration in the microbiota of 16p11.2^dp/+^ mice, marked by heightened levels of *Turicibacter* and *Prevotellaceae UCG_001*, and diminished levels of *Faecalibaculum* and *Romboutsia* (Figure 2C).

To gain preliminary insight into the functional changes in the gut microbial community of 16p11.2^dp/+^ group, we conducted a KEGG analysis using our 16S rRNA sequencing data. We began by identifying distinct KEGG orthologous markers from the differential taxa between the 16p11.2^dp/+^ and WT groups. These markers, representing functional bacterial genes, indicated variations in the functional capabilities of the gut microbiota between the two groups. We then mapped these markers to their associated metabolic pathways in the KEGG database. This revealed disruptions in the 16p11.2^dp/+^ group in several pathways, including amino acid metabolism, lipid metabolism, energy metabolism, glycan biosynthesis and metabolism, and xenobiotics biodegradation and metabolism. Additionally, we employed the *omixerRPM r-package (v0.3.2)* to realign KEGG orthologs with the annotation of Gut-Brain Modules (GBMs) (Lou et al., 2022). In keeping with the findings from KEGG, we identified a plethora of differential pathways from GBMs, belonging to amino acid metabolism, lipid metabolism, energy metabolism (Supplementary Figure S2F).

Taken together, the above results reveal substantial changes in the gut microbiota of 16p11.2^dp/+^ mice and underscore the predictive potential for disruptions in metabolic pathways using 16S rRNA data.

### Altered metabolic profile in 16p11.2^dp/+^ mice compared to WT mice

**Figure 3:**
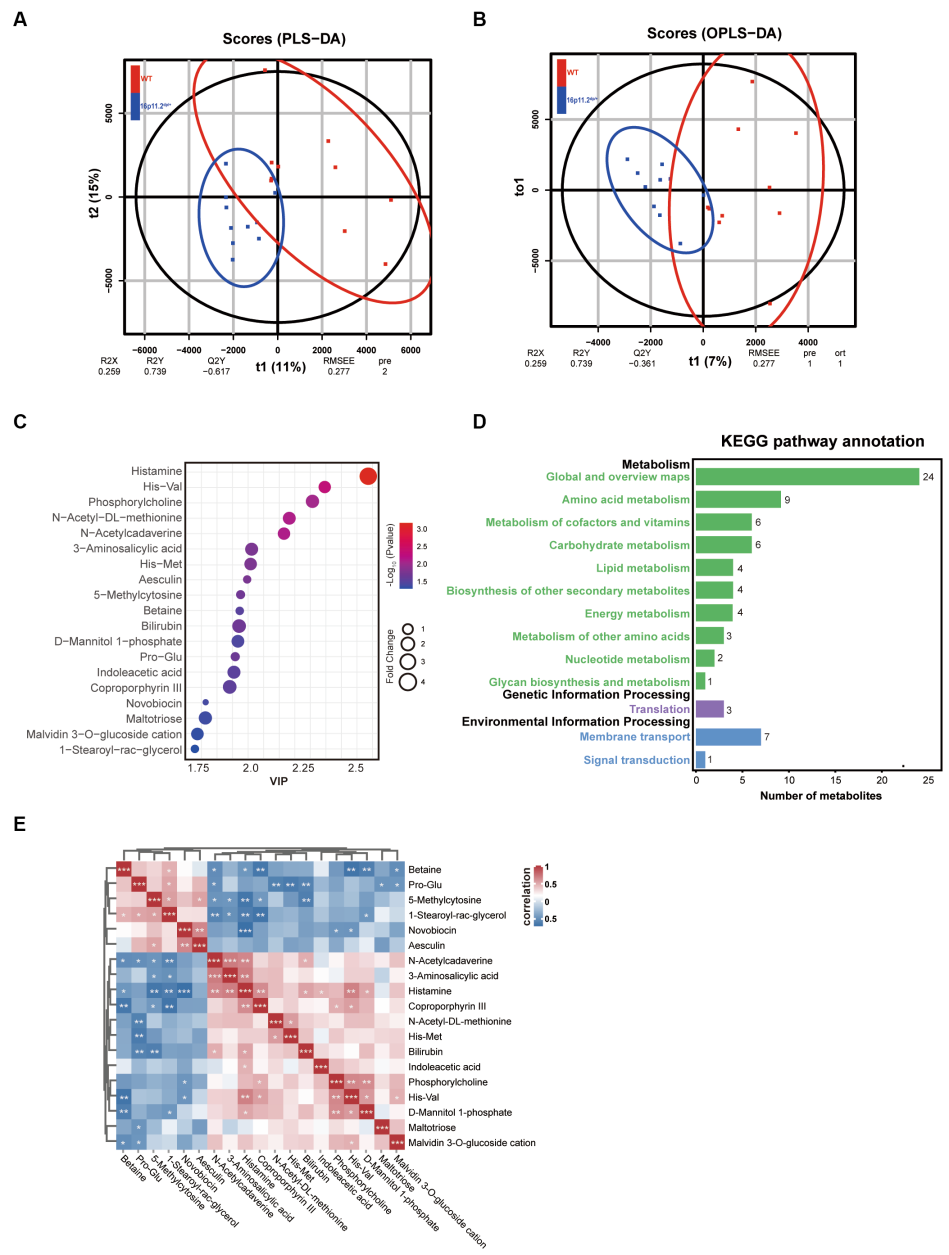
Altered gut microbial metabolic profile in 16p11.2^dp/+^ mice compared to WT mice. **(A)** Clustering analysis using partial least-squares discriminant analysis (PLS-DA). **(B)** Clustering analysis using orthogonal partial least-squares discriminant analysis (OPLS-DA). **(C)** Bubble chart representing 19 significantly different metabolites between 16p11.2^dp/+^ and WT groups. Metabolites with >1.5-fold changes, VIP ≥ 1, and p < 0.05 (t-test) are displayed. **(D)** Thirteen KEGG pathways exhibited significant differences between 16p11.2^dp/+^ and WT groups. **(E)** Spearman’s correlation analysis of significantly different metabolites, with red indicating a positive correlation and blue indicating a negative correlation. The intensity of color reflects the strength of the correlation. Statistical significance is represented as follows: Data is presented as mean ± SEM. ^*^p < 0.05, ^**^p < 0.01, ^***^p < 0.001, ns: not significant, n = 10 per group.

Microbial metabolites can impact the host’s physiology and behavior via various pathways, with some metabolites even entering the bloodstream. Hence, we explored the metabolic profile of the same samples as those used for the 16S rRNA analysis through LC/MS. The fecal samples from the 16p11.2^dp/+^ and WT groups were effectively distinguished based on the results of PLS-DA and OPLS-DA (Figures 3A,B). Subsequently, our metabolite profiling identified 19 significantly altered metabolites, with histamine showing the most substantial variations (Figure 3C and Supplementary Table S1).

To infer the potential functional changes in the gut microbiota of 16p11.2^dp/+^ mice, we conducted a KEGG analysis. We mapped the identified metabolites to their corresponding KEGG orthologous markers that represent the enzymes involved in the metabolic reactions. This analysis revealed significant differences in thirteen KEGG pathways between the two groups, spanning metabolism, genetic information processing, and environmental information processing. Notably, the category of metabolism exhibited the highest enrichment of significantly altered metabolites (Figure 3D).

Further, we conducted an in-depth exploration of the interrelationships of these metabolites through Spearman correlation analysis, which is visually depicted in a heatmap (Figure 3E). In summary, our metabolomics analysis unveiled 19 differentially regulated metabolites of significant relevance, and demonstrated enrichment in multiple KEGG pathways associated with metabolism.

### Alteration of neurotransmitter network activity primarily liked to histamine metabolism in 16p11.2^dp/+^ mice

**Figure 4:**
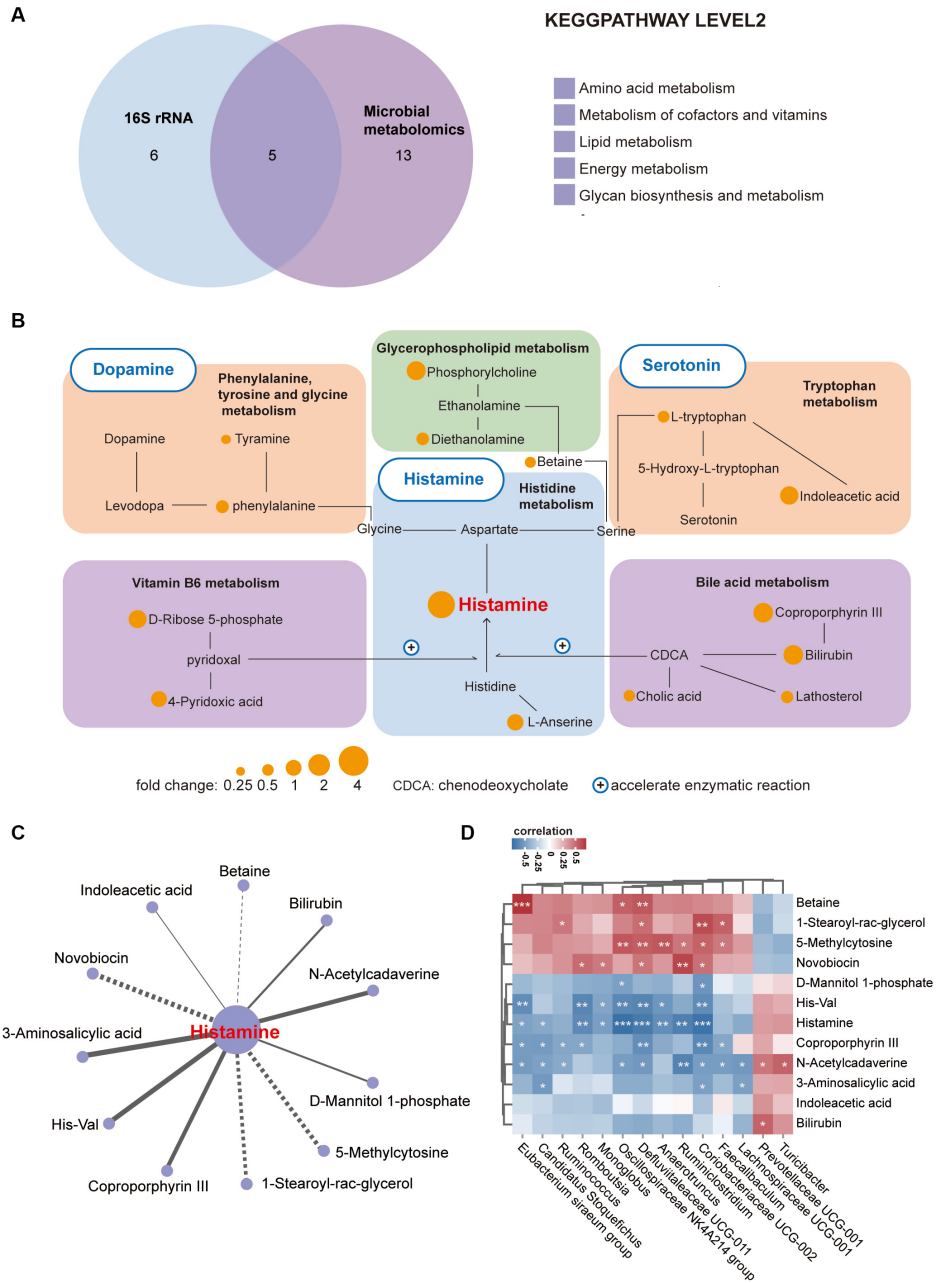
Enrichment of a neurotransmitter network centered on histamine in 16p11.2^dp/+^ mice. **(A)** Venn diagram illustrates the number of altered KEGG pathways shared between 16p11.2^dp/+^ and WT mice at the level 2. **(B)** Differentially expressed metabolites associated with the neurotransmitter network between the 16p11.2^dp/+^ and WT groups (Fold change >1.24, VIP ≥ 1, *p* < 0.1, t-test). **(C)** Network analysis linking histamine with significant differential metabolites. Lines connecting metabolites indicate the direction of their association with each genus of microbe, represented by solid lines (positive) or dotted lines (negative) (|r| > 0.4, *p* < 0.05). **(D)** Spearman’s correlation between differentially abundant species at the genus level, and metabolites associated with histamine metabolism. The correlation effect is indicated by a color gradient from blue (negative correlation) to red (positive correlation). Statistical significance is indicated as follows: Data are presented as mean ± SEM. ^*^p < 0.05, ^**^p < 0.01, ^***^p < 0.001, assessed using the t-test. n = 10 per group.

Given the intricate interplay between gut microbiota and intestinal metabolites, both of which frequently contribute to disease pathogenesis, we performed a comprehensive combined analysis of these factors. Initially, we conducted an integrated analysis of 16S rRNA and metabolomics data at the KEGG pathway Level 2, revealing intersections in five pathways (Figure 4A). Subsequently, while analyzing the distinctive metabolites linked to these five pathways, we revealed a neurotransmitter network involving histamine, 5-HT, and dopamine. Histamine primarily serves as the central hub within this network, with metabolic connections to other neurotransmitters (Figure 4B). Notably, vitamin B6 (Ferrante and Mercogliano, 2023) and bile acids (Ku, 2014) have the capacity to boost the enzymatic activity of histidine decarboxylase (HDC), a pivotal enzyme in histamine synthesis, and their metabolic profiles exhibit notable dynamism. Further, to identify metabolites and bacteria closely linked to histamine, we conducted a correlation analysis between histamine and other significantly variant metabolites, uncovering a profound association with metabolites like Coproporphyrin III and 1-Stearoyl-rac-glycerol (Figure 4C). Moreover, we delved into the relationships between metabolites and various bacterial genera at the genus level, notably *Coriobacteriaceae UCG_002*, *Defluviitaleaceae UCG_011*, and *Oscillospiraceae NK4A214 group*, thereby mapping their roles within the microbial community (Figure 4D). In summary, the disruptions observed in histamine metabolism in 16p11.2^dp/+^ mice are intricately linked with the metabolic activities of gut bacteria.

## Discussion

Our research reveals alterations in the gut microbiome and microbial neurotransmitter profiles linked to ASD traits. Comparing with wild-type mice, we observed reduced gut microbial diversity and specific shifts in microbial population abundances correlated with ASD-like behaviors. Moreover, our metabolomic analysis highlighted significant changes in metabolites related to amino acid pathways, further suggesting a potential link between the gut microbiota and ASD-like behaviors. These comprehensive results underscore the gut-brain axis’s role in ASD pathogenesis, offering new perspectives for future research.

Building on our findings, the 16p11.2^dp/+^ mouse model further illustrates ASD-like symptoms. This specific CNV, with a notable percentage of cases occurring *de novo* (Rein and Yan, 2020), is significantly linked to ASD, schizophrenia, and intellectual disability. Behavioral assessments revealed core ASD symptoms such as social deficits and repetitive behaviors, consistent with previous studies on this model (Rein et al., 2021). Moreover, our evaluation of open arm exploration time and performance in the elevated plus maze revealed no discernible changes in 16p11.2^dp/+^ mice. Previous studies have suggested that 16p11.2^dp/+^ mice may demonstrate a reduction in open arm time when tested in the elevated plus maze, but this effect may vary depending on gender (Bristow et al., 2020). Our study highlights the typical ASD-related features exhibited by the 16p11.2^dp/+^ microduplication and suggests that 16p11.2^dp/+^ mice could serve as a valuable model for ASD-related research.

To delve into the gut microbial implications under the overarching brain-related dysfunction, we employed 16S rRNA sequencing to investigate alterations in the gut microbiota composition of 16p11.2^dp/+^ mice. Utilizing α-diversity metrics like observed species richness, Chao1, ACE, and Shannon indices, alongside β-diversity analysis through PCoA, we observed a biodiversity decrease in the 16p11.2^dp/+^ mice, aligning with prior ASD studies (Gilbert et al., 2013; Golubeva et al., 2017; Kang et al., 2017; Lim et al., 2017; Kang et al., 2018; Sharon et al., 2019; Wang et al., 2019). Our analysis of phylum-level composition, particularly the *Firmicutes* and *Bacteroidetes*, showed variations that reflect the complex nature of gut microbiota dynamics observed in other studies, without specifying distinct trends (Tomova et al., 2015; Strati et al., 2017; Zhang et al., 2018). At the genus level, we observed an increase in *Turicibacter* and *Prevotellaceae UCG_001*, and a decrease in *Faecalibaculum* and *Romboutsia*. These observations align with prior studies of fecal microbiome analyses in autistic children, albeit with some nuances. For example, *Turicibacter* has been implicated in ASD-related behaviors, as demonstrated by symptom improvement in VPA mouse models following fecal microbiota transplantation (Wang J. et al., 2023). Additionally, research has shown an inverse correlation between *Turicibacter* abundance and sex hormones, potentially offering insights into the prevalence of ASD in females (Wu et al., 2022). In a high-fat diet-induced ASD mouse model, metformin was found to enhance *Faecalibacterium*, leading to a positive correlation with sociability and components of the 5-HT pathway (Deng et al., 2022a). *Prevotellaceae UCG_001* and *Faecalibaculum* are known to be associated with the presence of short-chain fatty acids (Kang et al., 2023), which have been implicated in the development of ASD (Mac Fabe, 2015). Given the limitations of 16S rRNA sequencing in fully characterizing the microbial community, we plan to conduct metagenomic analysis in the future to analyze at various taxonomic levels (such as species, genus, and phylum). This effort aims to identify specific species involved in the regulation of ASD-like behavior, thereby enhancing our understanding of the microbiome’s impact on ASD, and expanding upon the insights gained from our current research findings.

The potential influence of gut microbiota on ASD through the modulation of intestinal metabolites presents an intriguing area of investigation. In this study, we conducted a comprehensive analysis of fecal metabolites using LC/MS, identifying 19 metabolites with significant differences, notably histamine. These metabolites have diverse functions related to metabolism, genetic information processing, and environmental information processing. Remarkably, the most enriched pathway among these metabolites is amino acid metabolism, as indicated by KEGG analysis. We established the functional links between gut microbiota and metabolites in 16p11.2^dp/+^ mice. Through integrating KEGG analysis of 16S rRNA and metabolomics data, we unveiled an altered neurotransmitter network in 16p11.2^dp/+^ mice. This finding is in keeping with emerging evidence suggesting that children with ASD often exhibit irregular neurotransmitter levels, including histamine, serotonin, dopamine, and gamma-aminobutyric acid (GABA) (Montgomery et al., 2018; Strandwitz, 2018; De Palma et al., 2022; Montanari et al., 2022), known to be influenced by gut bacteria (Dinan and Cryan, 2017; Chen et al., 2021; Dicks, 2022). Our study revealed disruptions in the neurotransmitter network, with histamine being the most significantly altered core neurotransmitter among them.

Histamine, a pivotal neurotransmitter, has been implicated in neurological disorders (Nuutinen and Panula, 2010; Scammell et al., 2019), including ASD (Fernandez et al., 2012; Wright et al., 2017). Previous research has highlighted elevated blood histamine levels in children diagnosed with ASD (Rashaid et al., 2022). The effects of histamine primarily occur through its interaction with various histamine receptors, notably H1R, H2R, H3R, and H4R. Extensive literature suggests that targeting H2R in the brains of individuals with ASD and H3R in mouse brains shows promise in alleviating ASD symptoms (Linday, 1997; Linday et al., 2001; Eissa et al., 2020; Venkatachalam et al., 2021). Furthermore, histamine plays a role in enhancing the transmission of other neurotransmitters, including serotonin, norepinephrine, and dopamine in the brain (Flik et al., 2015). Histamine within the intestinal lumen has the potential to be absorbed into the bloodstream (Tidmarsh, 1932), where it can serve multiple functions, including the regulation of neurological disorders. Additionally, histamine is associated with various gastrointestinal disorders, such as irritable bowel syndrome (Barbara et al., 2004) and inflammatory bowel diseases (Dvornikova et al., 2023). Gut histamine can stimulates gastric acid secretion via H2R, induce abdominal discomfort through H4R activation (De Palma et al., 2022), and enhance intestinal motility (Mochizuki, 1953), leading to diarrhea (Smolinska et al., 2022), a common clinical symptom in ASD.

The level of histamine in the intestinal tract is maintained through a dynamic equilibrium that involves its synthesis, degradation, absorption, and secretion processes. Gut bacteria can influence histamine levels in feces by regulating histamine secretion (Sanchez-Perez et al., 2022), expediting synthesis process via HDC (Lucas et al., 2008) and promoting degradation through histamine N-methyltransferase (HNMT) and diamine oxidase (DAO) (Shi et al., 2022). Our analysis of the correlation between histamine and bacterial species revealed an array of bacteria distinct from those previously reported to influence histamine, either through production [e.g., *Lactobacillus*, *Enterococcus*, *Klebsiella* (Krell et al., 2021; De Palma et al., 2022)] or by affecting histamine levels [e.g., *Pseudomonas aeruginosa*, *Escherichia coli* (Fiorani et al., 2023)]. Therefore, we postulate that the augmented levels of histamine in the intestinal cavity of 16p11.2^dp/+^ mice are less likely to be significantly influenced by those known histamine-influencing microbiomes, but more likely attributed to host synthesis and/or secretion. Additionally, our GBMs analysis enabled the identification of histamine degradation pathways within samples from both groups under study. However, due to the insufficient sequencing depth of 16S rRNA, as well as the still-evolving GBMs, precision of the analysis was compromised. Even though the statistical values of histamine degradation pathways in the 16p11.2^dp/+^ group showed a noticeable decline compared to the WT group, it did not culminate in a statistically significant difference (Supplementary Table S2). In this send, it is possible that altered histamine metabolism might influenced the abundance of some, if not all, bacteria identified in our study. Intriguingly, the levels of these bacteria were inextricably linked to a plethora of bioclinical implications, including autistic symptom (Ye et al., 2021; Deng et al., 2022b), intestinal inflammation (Gu et al., 2022), gut neurotransmitter production (Deng et al., 2022b; Wang Z. J. et al., 2023), and bile acid metabolism (Jian et al., 2022).

Based on these findings, we propose that the 16p11.2 CNV is closely associated with which are manifested as increased intestinal histamine levels, imbalances in gut microbiota and metabolites, potentially contributing to the development of ASD. Emerging evidence indicates that the genes MAPK3 and MAZ, located in the 16p11.2 region, are implicated in the control of degranulation in intestinal mast cells, leading to histamine release (Bilotta et al., 2021; Gou et al., 2023). Elevated histamine levels in the lumen have the potential to induce intestinal inflammation by triggering mast cell activation (De Palma et al., 2022), potentially resulting in its degranulation. Future research warrants the investigation on (1) the potential morphological and molecular changes of mast cells resulting from defects in 16p11.2-associated genes; (2) the impact of these changes on specific gut microbiomes and metabolism; (3) the causal mechanisms linking histamine to 16p11.2-driven autistic behaviors; and (4) adopting metagenomic sequencing for future material reanalysis and annotation.

## Conclusion

In conclusion, our investigation has unveiled the manifestation of gut dysbiosis in 16p11.2^dp/+^ mice across diverse taxonomic levels. Notably, these mice exhibit diminished microbial diversity and a perturbed structure within their fecal microbiota. Furthermore, our integrated analysis of gut microbiota and metabolites has elucidated the aberrant feature of a predominant neurotransmitter network primarily centered around histamine. These findings provide novel insights into the pathogenesis and potential therapeutic avenues for 16p11.2 CNV-associated ASD, with a particular focus on targeting gut neurotransmitter metabolism.

## Data availability statement

The raw data of the 16S rRNAseq is available at https://www.ncbi.nlm.nih.gov/bioproject/PRJNA1056406. The microbial metabolomics data presented in the study are included in the Supplementary material, further inquiries can be directed to the corresponding author.

## Ethics statement

The experimental procedures involving animals were all approved by the Institutional Animal Care and Use Committee at the Southern University of Science and Technology (SUSTC-2019-174). The study was conducted in accordance with the local legislation and institutional requirements.

## Author contributions

ZF: Conceptualization, Data curation, Formal analysis, Writing – original draft, Writing – review & editing. XY: Conceptualization, Data curation, Writing – review & editing. YJ: Conceptualization, Formal analysis, Writing – review & editing. XinlM: Methodology, Software, Supervision, Validation, Visualization, Writing – review & editing. HuaL: Methodology, Software, Visualization, Writing – review & editing. YY: Methodology, Software, Visualization, Writing – review & editing. JC: Methodology, Software, Visualization, Writing – review & editing. ZC: Methodology, Software, Visualization, Writing – review & editing. HuiL: Data curation, Project administration, Writing – review & editing. XZ: Data curation, Project administration, Writing – review & editing. XinjM: Supervision, Validation, Writing – review & editing. NL: Supervision, Validation, Writing – review & editing, Funding acquisition. DW: Supervision, Validation, Writing – review & editing. JJ: Supervision, Validation, Writing – review & editing.

## Footnotes

^1^ https://xcmsonline.scripps.edu
