# Supplementary material for: Microbiota profiling reveals alteration of gut microbial neurotransmitters in a mouse model of autism-associated 16p11.2 microduplication
Source: Front Microbiol. 2024 Mar 26;15:1331130. doi: 10.3389/fmicb.2024.1331130 (PMC11002229; doi:10.3389/fmicb.2024.1331130)
Supplement: Supplementary file 1 [file Data_Sheet_1.docx]

Supplementary Material

**
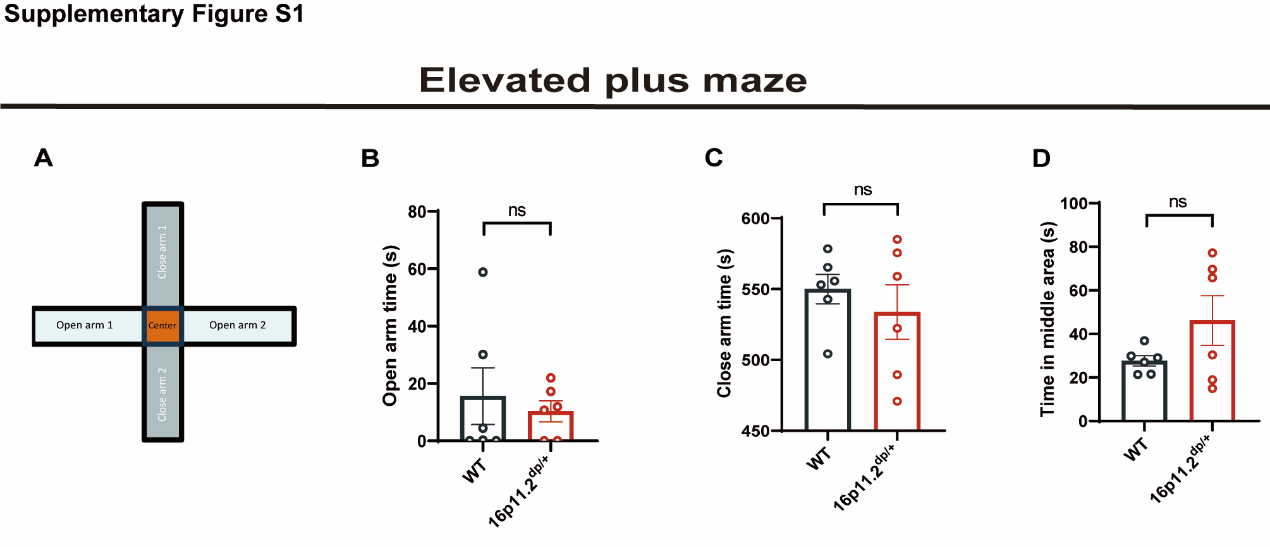
**

**Supplementary Figure S1: Absence of anxiety-like behavior in 16p11.2^dp/+^ mice**

(A) Illustration depicting the elevated plus-maze test setup. (B-D) 16p11.2^dp/+^ mice did not exhibit statistically significant differences in the open arm, closed arm, or central area compared to WT mice., with each group comprising 6 mice. Data is presented as mean ± SEM, with "ns" indicating non-significant findings.


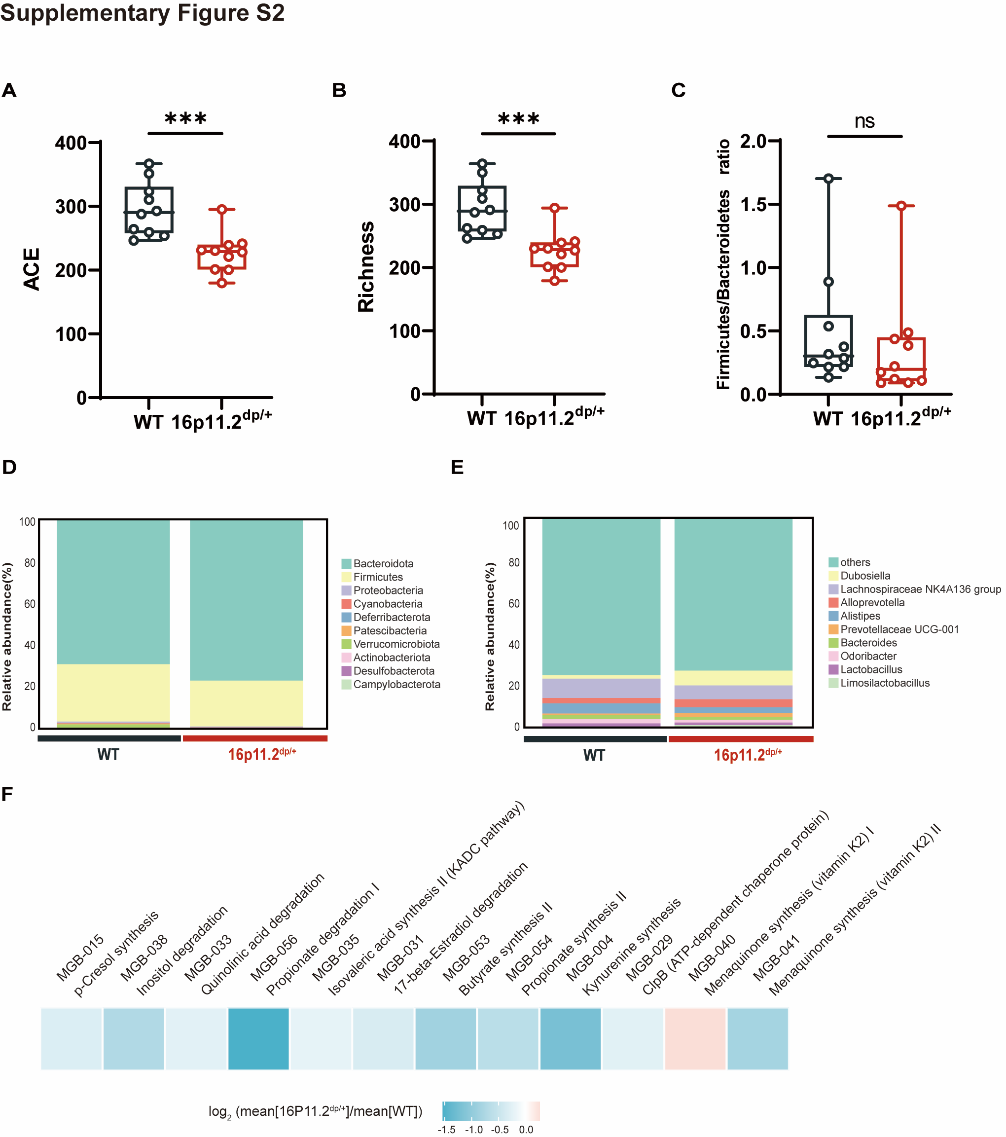


Revised Supplementary FigureS2. Microbiota analysis in 16p11.2^dp/+^ mice.

(A-B) Alpha diversity evaluated using the ACE index (A) and the Richness index (B) in fecal samples collected from 16p11.2^dp/+^ mice and WT mice. (C) The Firmicutes/Bacteroidetes ratio in 16p11.2^dp/+^ and WT mice. (D) Relative abundance of phylum genera between 16p11.2^dp/+^ and WT mice. (E) Relative abundance of genus genera between 16p11.2^dp/+^ and control group. (F) Heat map showing the significant changes in microbial metabolic functions annotated as GBM (based on Mann-Whitney test, P< 0.05, detailed in the revised Supplemental TablesS2). Statistical significance is denoted as follows: Data is presented as mean ± SEM. ***P < 0.001, ns: not significant, Subfigures A, B, C, D and E assessed using the T-test. n = 10 per group.

**Supplementary Table S1**

|  |  |  |  |
| --- | --- | --- | --- |
| **Metabolites** | **P-value** | **Fold change(16p11.2^dp/+^/WT)** | **VIP** |
| 1-Stearoyl-rac-glycerol | 0.04969642 | 0.531074399 | 1.70293 |
| 3-Aminosalicylic acid | 0.0168953 | 1.74597906 | 2.02295 |
| 5-Methylcytosine | 0.02003519 | 0.625646993 | 1.96064 |
| Aesculin | 0.01740032 | 0.518551382 | 1.9978 |
| Betaine | 0.03073245 | 0.536889779 | 1.95577 |
| Bilirubin | 0.02083466 | 2.040625434 | 1.95233 |
| Coproporphyrin III | 0.02795119 | 2.02624479 | 1.89928 |
| D-Mannitol 1-phosphate | 0.03617493 | 1.651881158 | 1.94552 |
| His-Met | 0.01653443 | 1.584035001 | 2.01724 |
| Histamine | 0.00066629 | 4.123029616 | 2.68083 |
| His-Val | 0.00489682 | 1.522842572 | 2.43529 |
| Indoleacetic acid | 0.02949105 | 1.753212952 | 1.92319 |
| Maltotriose | 0.04250159 | 1.84751221 | 1.762 |
| Malvidin 3-O-glucoside cation | 0.04693437 | 1.587499006 | 1.7171 |
| N-Acetylcadaverine | 0.00750421 | 1.720804839 | 2.2064 |
| N-Acetyl-DL-methionine | 0.00774959 | 1.538344369 | 2.23631 |
| Novobiocin | 0.0401346 | 0.385962333 | 1.76354 |
| Phosphorylcholine | 0.00904199 | 1.884575452 | 2.36604 |
| Pro-Glu | 0.02206644 | 0.645213747 | 1.93274 |

Significant differential metabolites: P<0.05, Fold change>1.5or<0.67, VIP>1.

**Supplementary Table S1**

Comprehensive information on significant differential metabolites, comprising P-value, fold change, and VIP values.

**Supplementary Table S2**

| Module | Description | meanWT | meanASD | P value | LOG2(FC) |
| --- | --- | --- | --- | --- | --- |
| MGB015 | p-Cresol synthesis | 14672.273 | 11684.043 | 0.035463 | -0.3285528 |
| MGB037 | Inositol synthesis | 12045.239 | 12002.293 | 0.684211 | -0.005153 |
| MGB016 | p-Cresol degradation | 2.683 | 0.767 | 0.084068 | -1.8065486 |
| MGB038 | Inositol degradation | 2601.034 | 1567.451 | 0.035463 | -0.7306649 |
| MGB039 | g-Hydroxybutyric acid (GHB) degradation | 355.217 | 234.734 | 0.739364 | -0.5976738 |
| MGB033 | Quinolinic acid degradation | 26434.718 | 21998.218 | 0.035463 | -0.2650473 |
| MGB055 | Propionate synthesis III | 13243.698 | 12799.2865 | 0.578742 | -0.0492426 |
| MGB034 | Isovaleric acid synthesis I (KADH pathway) | 3485.969 | 6732.1725 | 0.247451 | 0.9495124 |
| MGB056 | Propionate degradation I | 11.325 | 3.9 | 0.002999 | -1.537965 |
| MGB035 | Isovaleric acid synthesis II (KADC pathway) | 21610.8115 | 18407.886 | 0.035463 | -0.2314293 |
| MGB036 | S-Adenosylmethionine (SAM) synthesis | 27483.916 | 23071.985 | 0.063013 | -0.2524454 |
| MGB051 | Glutamate degradation II | 1.067 | 0 | 0.086687 | #NUM! |
| MGB052 | Butyrate synthesis I | 10723.379 | 9052.467 | 0.352681 | -0.2443767 |
| MGB031 | 17-beta-Estradiol degradation | 26357.913 | 20402.496 | 0.043257 | -0.3694905 |
| MGB053 | Butyrate synthesis II | 3105.5475 | 1693.8545 | 0.028806 | -0.8745377 |
| MGB032 | Quinolinic acid synthesis | 18295.1 | 16319.201 | 0.217563 | -0.1648869 |
| MGB054 | Propionate synthesis II | 2288.558 | 1466.842 | 0.035463 | -0.6417254 |
| MGB010 | Histamine degradation | 0.633 | 0.167 | >0.999999 | -1.9223574 |
| MGB050 | Glutamate degradation I | 208.949 | 113.867 | 0.052426 | -0.8758012 |
| MGB019 | GABA degradation | 775.637 | 1069.617 | 0.684211 | 0.46364077 |
| MGB004 | Kynurenine synthesis | 212.592 | 91.383 | 0.023231 | -1.2180896 |
| MGB048 | Propionate synthesis I | 18.7835 | 76.567 | 0.362272 | 2.02725682 |
| MGB026 | Nitric oxide synthesis II (nitrite reductase) | 0 | 0.2 | >0.999999 | #DIV/0! |
| MGB005 | Tryptophan synthesis | 19567.726 | 15687.251 | 0.052426 | -0.3188835 |
| MGB027 | Nitric oxide degradation I (NO dioxygenase) | 477.837 | 379.217 | 0.314999 | -0.3334949 |
| MGB049 | Tryptophan degradation | 3258.922 | 2060.667 | 0.063013 | -0.6612834 |
| MGB006 | Glutamate synthesis I | 35881.212 | 31573.81 | 0.063013 | -0.1845003 |
| MGB028 | Nitric oxide degradation II (NO reductase) | 19.2 | 0.4 | 0.173375 | -5.5849625 |
| MGB007 | Glutamate synthesis II | 26983.373 | 23765.9385 | 0.075256 | -0.1831753 |
| MGB029 | ClpB (ATP-dependent chaperone protein) | 25884.468 | 21391.768 | 0.035463 | -0.2750309 |
| MGB022 | GABA synthesis III | 10828.199 | 10654.243 | 0.684211 | -0.0233652 |
| MGB044 | Acetate synthesis II | 27676.986 | 22796.962 | 0.190316 | -0.2798453 |
| MGB023 | Dopamine degradation | 8.648 | 13.267 | 0.402574 | 0.61740375 |
| MGB045 | Acetate synthesis III | 13859.893 | 17906.133 | 0.970512 | 0.36953769 |
| MGB024 | DOPAC synthesis | 9092.0595 | 8112.15 | 0.314999 | -0.1645228 |
| MGB046 | Acetate synthesis IV | 6.05 | 2.2 | 0.14301 | -1.4594316 |
| MGB047 | Acetate degradation | 9952.002 | 10535.175 | 0.481251 | 0.0821556 |
| MGB025 | Nitric oxide synthesis I (NO synthase) | 0.82 | 0 | 0.210526 | #NUM! |
| MGB040 | Menaquinone synthesis (vitamin K2) I | 10900.4945 | 13090.793 | 0.035463 | 0.26415891 |
| MGB041 | Menaquinone synthesis (vitamin K2) II | 2550.234 | 1430.45 | 0.028806 | -0.8341606 |
| MGB020 | GABA synthesis I | 20.992 | 7.467 | 0.088095 | -1.491239 |
| MGB021 | GABA synthesis II | 33.417 | 21.767 | 0.224642 | -0.6184396 |
| MGB043 | Acetate synthesis I | 25544.711 | 22820.535 | 0.314999 | -0.162692 |

Summary of the statistical results of the 43 GBMs between the two groups.
